# Variability of Urinary Creatinine in Healthy Individuals

**DOI:** 10.3390/ijerph18063166

**Published:** 2021-03-19

**Authors:** Gerd Sallsten, Lars Barregard

**Affiliations:** Occupational and Environmental Medicine, School of Public Health and Community Medicine, Institute of Medicine, Sahlgrenska Academy, University of Gothenburg, SE-40530 Gothenburg, Sweden; lars.barregard@amm.gu.se

**Keywords:** biomonitoring, variability, creatinine, specific gravity, 24 h urine, spot urine, biobank

## Abstract

Many urinary biomarkers are adjusted for dilution using creatinine or specific gravity. The aim was to evaluate the variability of creatinine excretion, in 24 h and spot samples, and to describe an openly available variability biobank. Urine and blood samples were collected from 60 healthy non-smoking adults, 29 men and 31 women. All urine was collected at six time points during two 24 h periods. Blood samples were also collected twice and stored frozen. Analyses of creatinine in urine was performed in fresh urine using an enzymatic method. For creatinine in urine, the intra-class correlation (ICC) was calculated for 24 h urine and spot samples. Diurnal variability was examined, as well as association with urinary flow rate. The creatinine excretion rate was lowest in overnight samples and relatively constant in the other five samples. The creatinine excretion rate in each individual was positively correlated with urinary flow rate. The creatinine concentration was highest in the overnight sample and at 09:30. For 24 h samples the ICC was 0.64, for overnight samples it was 0.5, and for all spot samples, it was much lower. The ICC for urinary creatinine depends on the time of day of sampling. Frozen samples from this variability biobank are open for researchers examining normal variability of their favorite biomarker(s).

## 1. Introduction

Biomarkers of exposure or biomarkers of effects are often used in occupational health surveillance and in epidemiological research. For most biomarkers there is, however, limited information about normal day-to-day variability within individuals in the general population. Therefore, there is also scarce information about the relation between within- and between-individual variability. In occupational settings variability in biomonitoring data has been presented for a number of substances [1]. For urinary biomarkers there may be diurnal variability making it important to fix the time of day the sampling is performed, but again there is very limited information in the literature about diurnal variability. This is also true when biomarkers in urine are adjusted for dilution using creatinine or specific gravity. In addition, variability of creatinine excretion in timed samples has rarely been studied and the relation between creatinine excretion and urinary flow rate is unclear [2,3]. Variability of creatinine excretion is of special interest since it is commonly used to “normalize” concentrations of biomonitoring results in urine spot samples which otherwise are greatly affected by varying diuresis [4].

We have established a “variability biobank” based on a convenience sample of 60 healthy adult non-smoking individuals from Gothenburg, Sweden to make it possible for us and other researchers to examine normal variability of various biomarkers. Blood and urine sampling (timed samples over 24 h) was performed twice and more than 20,000 aliquots from these individuals are now stored frozen (−80 °C). This paper describes the within- and between-individual variability of creatinine excretion in 60 healthy individuals, both the diurnal variability (in spot samples) and variability in 24 h samples. In addition, we describe in detail the types of sample collected and stored as well as background factors for these individuals, including diurnal variation in urine volume, flow rate, and specific gravity.

## 2. Materials and Methods 

### 2.1. Participants and Background Information

The study includes a convenience sample of 60 healthy, non-smoking subjects 21–64 years (31 women and 29 men) without diabetes, hypertension, kidney disease (based on medical history), or self-reported medications for other chronic diseases. Fifty participants were recruited in 2012 and another 10 in 2013, mainly from employees or students at the Sahlgrenska Hospital, the University of Gothenburg, or personal contacts. In total 82 individuals were asked to participate, 11 refused and 11 did not fulfill the criteria. The participants filled in a short questionnaire about country of birth, height and weight, consumption of fish, meat and rice (times/week), and being vegetarian or vegan. In addition, there was a question about presence of amalgam fillings, leisure time exercise (at least 30 min twice a week, yes/no) and a physically demanding job (yes/no). The study protocol was approved by the Ethics Committee at the University of Gothenburg (Dnr: 213-12, date: 20120405), and informed consent was obtained from all study participants. The participants received no monetary incentive.

### 2.2. Blood and Urine Sampling

Complete 24-h urine samples were collected at specified times of the day in order to study the circadian rhythm. This was repeated twice with an interval of approximately one week. In addition, blood samples were taken on the same days as the urine samples. Sampling was performed during weekdays at home or at the workplace. The participants had no food or drink restrictions during the study.

The urine samples were collected in six 1 L (maximum volume 1070 mL) high-density polyethylene bottles with polypropylene screw caps (Bibby Sterling LTD, Staffordshire, UK) and no preservatives. The participants were instructed to urinate at six fixed times (9:30, 12:00, 14:30, 17:30, 22:00, and first morning). On the morning of the starting day, upon rising, participants were instructed to discharge the first void of the day and record the date and time in a separate protocol as the starting point for the 24 h urine sampling. They were instructed to collect each void, at the specific time, using a separate bottle and to record the time of each void. If they needed to urinate in between the fixed times they used the next bottle and then filled it again at the specified time point. In the next morning they collected the first void of the day, representing the overnight (ON) urine sample (from approximately 22:00 until the first morning void). Participants were instructed to store their urine samples in a refrigerator and return all samples on the day they had completed the collection. All participants provided urine samples with a mean 24-h volume above 800 mL and a total sampling time above 20 h, except for one participant who missed part of the overnight sample on one of the two days. However, the total sampling time in this case was 18 h. One subject collected all urine from 14:30 to 22:00, skipping the fixed time at 17:30, but the 24-h (24 h) sampling for this person was still complete. 

Non-fasting blood samples were collected twice (at about 9 a.m.), in connection with the two 24 h urine samplings. Each time, approximately 24 mL blood was drawn into four different tubes.

### 2.3. Sample Processing and Laboratory Measurements

All urine samples were transferred to 2 mL polypropylene micro tubes (Sarstedt, Nümbrecht, Germany). From each urine bottle 30 tubes were filled with 1.75 mL urine in each, in total 180 tubes per subject each day (in total 360 tubes from each individual for the two 24-h sampling periods). For each urine sample the total volume was recorded and collection times were checked. All these samples (apart from the aliquot for creatinine, see below) were stored within 8 h in a freezer (−80 °C). The biobank thus includes in total nearly 21,500 tubes filled with urine.

Creatinine in urine is relatively stable [5] and, therefore, we expected no change in concentrations during the short transport times. Analyses of creatinine (U-Crea) in urine were performed in fresh urine transferred directly into tubes from each bottle, kept at 4 °C until analysis within three days of collection, using the Roche Creatinine enzymatic assay on the Cobas 6000 instrument (Roche Diagnostics Scandinavia AB, Solna, Sweden) with a limit of detection (LOD) of 0.01 g/L, and traceable to the International Standardization with isotope dilution mass-spectrometry (IDMS). These analyses were performed at the Sahlgrenska University hospital. In each run quality control samples were included. The laboratory takes part in extensive quality assurance programs (e.g., the “Equalis” program) for creatinine in both urine and serum, and it is an accredited laboratory for these analyses. The average deviation from the target value over nine analytical rounds was 0.48%. Imprecision (coefficient of variation), as calculated by duplicate analyses, was 1.4–3.6%. Specific gravity (SG) was measured with a Ceti Digit 012 refractometer (Medline, Oxfordshire, UK). 

Excretion rates of creatinine per hour and per 24 h were calculated from urinary concentrations, volumes, and sampling times. Urinary flow rates (UF) were calculated from urinary volumes and sampling times. 

Blood samples were collected in four different vacuum tubes, one containing Na-citrate (for plasma), one tube for serum, and two Li-heparin tubes for metal analyses, one with whole blood, and the other for collection of plasma and erythrocytes. Whole blood, plasma, serum, and erythrocytes were transferred to polypropylene (PP) tubes (0.2 mL or 2 mL). Information about brands of the blood collection tubes, type of pipettes used, centrifugation, volume stored in each PP tube and the total number of PP tubes for each collection time can be found in Table S1. In total there are nearly 3500 PP-tubes from the blood sampling stored (−80 °C) in the biobank. 

Analyses of creatinine in serum (S-Crea) were performed in fresh samples, kept at 4 °C until analysis, using the Roche Creatinine enzymatic assay on a Cobas 6000 instrument (Roche Diagnostics Scandinavia AB, Solna, Sweden) with an LOD of 5 µmol/L (0.57 mg/L). The analyses were performed at the Sahlgrenska University hospital with quality control as described for urinary creatinine. Estimated glomerular filtration rate (eGFR) in mL/min/1.73 m^2^ was calculated with the CKD-EPI equation [6].

### 2.4. Statistics

Descriptive statistics were calculated for background characteristics and for 24 h urinary volume, urinary flow rate and urinary excretion of creatinine after calculating the individual arithmetic means of the two 24 h sampling periods. The circadian rhythms of urine flow rate, SG and creatinine excretion were studied by calculating means and medians of individual arithmetic means (of the two days) for each time point on untransformed data, and differences between sex and time points were assessed using mixed models, see below. Associations between urinary flow rate and creatinine excretion rate were assessed within individuals by calculation of Spearman correlation coefficient (r_s_) for each participant. Overall mean of the correlation coefficients were then calculated. 

The number of urine samples very diluted or concentrated were calculated, since these are assumed not to give reliable results. Frequently used cut off values for creatinine are <0.3 g/L and >3 g/L [7,8] and for specific gravity <1.010 and >1.030 [9].

Since multiple samples were available for each study participant, the within (σ^2^_w)_ and between (σ^2^_bY_) individual variability were calculated using mixed-effects models, separately for creatinine excretion, creatinine concentration and urinary flow rate. Natural log-transformation was used since the data were highly skewed. To determine if common fixed mean exposure levels and common variances could be used for men and women (i.e., if µ_Y(men)_ = µ_Y(women)_, σ^2^_bY(men)_ = σ^2^_bY(women)_, and σ^2^_wY(men)_ = σ^2^_wY(women)_), we used mixed-effects models containing intercept and gender as fixed effects. Three different variance structures were compared: common between- and within-individual variances for men and women, distinct between-individual but common within-individual variances, and distinct between- and within-individual variances, using a likelihood ratio test (significance level; *p* < 0.05) where the difference in −2 log likelihood follows a chi square distribution [10]. The estimated ratio of the between-individual variance to total observed variance, the ICC (intra-class correlation coefficient), was calculated. In some models for creatinine (using the variance structure determined above) collection time or urinary flow rate (log-transformed) was also added as a fixed effect to evaluate the significance of these parameters (*p* < 0.05). When examining the association between urinary flow rate and creatinine excretion rate, also age and body mass index (BMI) were included in the model as fixed effects.

All calculations were performed with version 9.4 of the SAS software package (SAS Institute, Cary, NC, USA). Statistical significance was set at *p* < 0.05 for all tests except where otherwise stated, and two-sided confidence intervals were used.

## 3. Results

### 3.1. Study Population

The mean age of the study participants was 34 years (range 21–64), Table 1. Most participants were born in Sweden but 22 subjects were born in other countries (Iran, Iraq, USA, Russia, Finland, South Korea, Kyrgyzstan, Ukraine). Eight subjects were former smokers and the others were never smokers. Nearly 80% reported exercise for at least 30 min twice a week. One woman was vegetarian. Men and women had similar fish consumption but men consumed rice and meat more often. More men than women had amalgam fillings, Table 1. All individuals had serum creatinine and eGFR within normal limits. The mean serum creatinine was 76 µmol/L (range 49–116). The mean eGFR was 116 (range 64–156) mL/min/1.73 m^2^.

### 3.2. Urinary Flow Rate, Creatinine and Specific Gravity in 24 h Urine

The median urine volume in the 24 h samples was 1.6 L (range 0.84–3.7 L) and the median urinary flow rate (UF) was 66 mL/h, similar for men and women, but the flow rate per kg body weight was significantly higher in women than in men, Table 2 and Table 3. The 24 h creatinine excretion was significantly lower in women than in men (medians 1.26 g vs. 1.94 g) as was the creatinine concentration (medians 0.76 g/L vs. 1.26 g/L, and specific gravity (medians 1.014 vs. 1.019). 

The ICC for log-transformed creatinine concentration per 24 h was 0.72 and ICC for log-transformed UF was 0.62, Table 3. The same within- and between-individual variability could be used for men and women in these models (according to likelihood ratio test). Somewhat lower ICC was found for the creatinine excretion rate (0.64). The within-individual variability can also be expressed as the coefficient of variation (CV) [2]. The CV for 24 h creatinine excretion was 13%. There was a high correlation between specific gravity and creatinine concentration (r_s_ = 0.87 for the first day and r_s_ = 0.90 for the second day, both *p* < 0.001), Figure S1.

### 3.3. Diluted or Concentrated Urine Spot Samples

The numbers of very low (<0.3 g/L) or very high (>3 g/L) creatinine concentrations were only a few percent of all 718 samples (4.0% < 0.3 g/L, 2.2% > 3 g/L), Table S2. The highest fraction of low creatinine concentrations was found at time 12:00 (12.5%). 

With respect to specific gravity (SG) the fraction of samples outside the recommended limits [9] was higher (14.8% < 1.010, and 7.5% > 1.030). However only 10 samples (1.4%) were at or below 1.005 and none below 1.004. 

As expected, creatinine concentrations <0.3 g/L were more common in women than in men (6.5% vs. 1.4%) and U-Crea > 3 g/L was more common in men (3.5% vs. 1.1%). In women, very few SG samples were below 1.010, whereas it was the case in 10% of the men. About 12% (men) and 7% (women) of the samples had SG > 1.030 (data not shown).

### 3.4. Circadian Rhythm 

**Figure 1:**
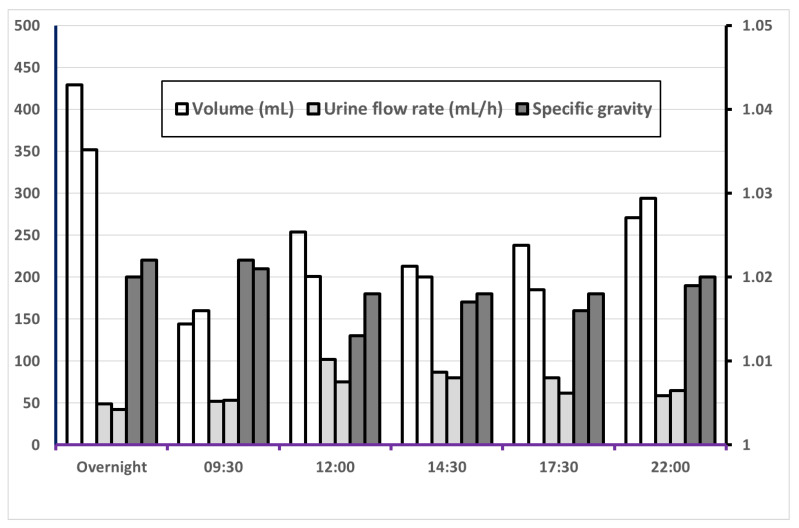
Median values of volume, urinary flow rate and specific gravity (on the right axis) for 60 subjects in each of two 24 h sampling periods. For each measure, the left bar represents Day 1 and the right bar Day 2.

**Figure 2:**
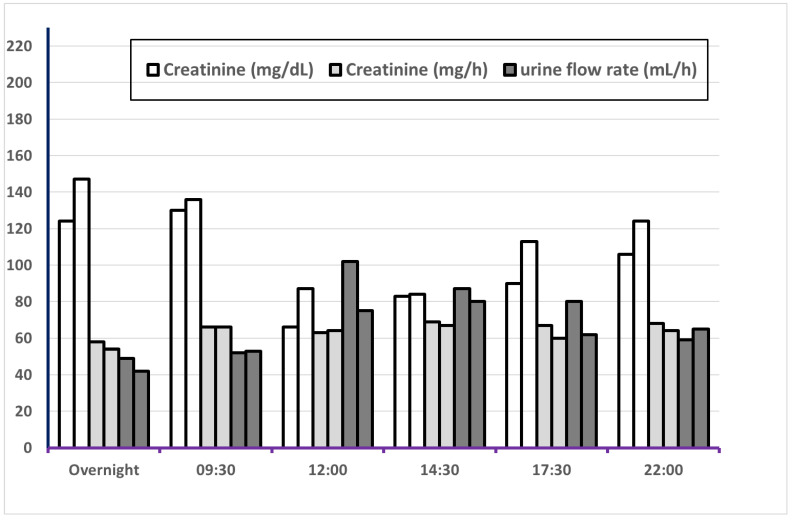
Median values of creatinine concentration, creatinine excretion rate and urine flow rate for 60 subjects on each of two 24 h periods. For each measure, the left bar represents Day 1 and the right bar Day 2.

Figure 1 and Figure 2 show the variation of volume, UF, SG, creatinine concentration and creatinine excretion rate over the six fixed sampling times. The largest urine volume was found in the overnight (ON) sample and at 22:00 because of longer sampling times on these occasions, Figure 2 and Table S3. Urinary flow rate (mL/h) was lowest in ON samples and highest at 12:00 and 14:30. As expected, specific gravity showed an opposite pattern with somewhat higher SG for overnight samples and at 09:30. The pattern was generally similar for men and women, Figure S2. The creatinine excretion rate was similar for most of the sampling times but lowest for ON, with statistically significant differences versus all other sampling time points. The creatinine concentration was, however, highest in the ON sample and at 09:30 with a similar pattern for men and women, Figure S3. Creatinine concentrations at these two sampling times were significantly higher than in samples from 12:00, 14:30, and 17:30. 

**Figure 3:**
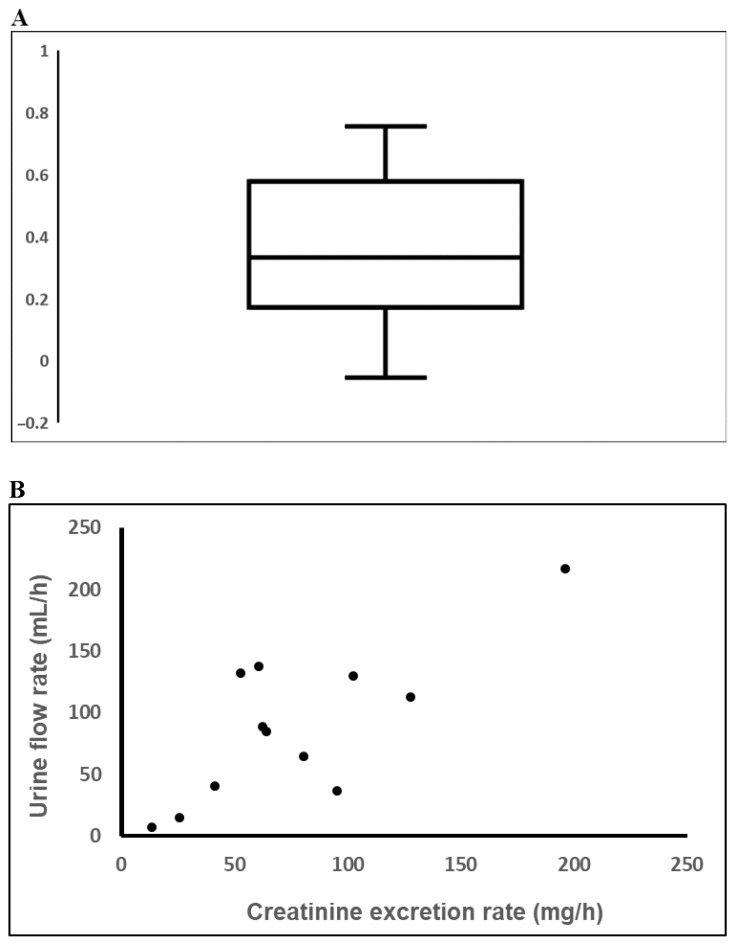
(**A**) Distribution of individual Spearman correlation coefficients for the association between creatinine excretion rate and urinary flow rate in 60 individuals. The box plot shows the 10th, 25th, 50th, 75th, and 90th percentiles. (**B**) The relationship for 12 spot samples in one individual (r_s_ = 0.55).

There was a significant positive association between urinary flow rate and creatinine excretion rate within individuals. The mean of the 60 individual Spearman correlation coefficients was 0.35, Figure 3A (*p* < 0.001, *t*-test). A somewhat lower correlation (mean r_s_ = 0.26), but still statistically significant (*p* < 0.001) was found when overnight samples were excluded. Figure 3B shows a typical association between urinary flow rate and creatinine excretion rate in one individual. The slope and intercept of a log-log regression of creatinine excretion rate (g/h) versus flow rate (mL/h) was for all samples (*n* = 718) 0.24 (*p* < 0.001) and –3.77, respectively, and without overnight samples (*n* = 599) 0.22 (*p* < 0.001) and –3.77, respectively. As mentioned in the method section, these results were obtained in mixed model analyses, adjusted for age, sex and BMI.

The ICC for log transformed creatinine excretion rate among all spot samples (*n* = 718) was 0.23, Table 3. The highest ICCs were found for ON samples and at 12:00 with ICC = 0.48 and 0.42, respectively. In these models similar within- and between-individual variability could be used for men and women, but this was not the case for urinary flow rate and creatinine concentration in spot samples (likelihood ratio test). For ON samples the ICC for creatinine concentration was 0.71 for women and 0.51 for men. The ICC for creatinine in spot samples from all sampling times were generally much lower than for ON samples. The creatinine excretion rate and the creatinine concentration were higher among men, Table 3, Table S4 (women) and Table S5 (men). For specific gravity the ICC for all 718 spot samples was 0.35 (data not shown).

## 4. Discussion

### 4.1. Urine Flow Rates 

Urinary volumes and flow rates were in agreement with the literature [11]. The urinary flow rate was lowest in overnight samples, which is a natural consequence of lower fluid intake during the night and in agreement with previous studies [3,12]. 

### 4.2. The 24 h Creatinine Excretion 

The mean 24 h creatinine excretion of creatinine (1.27 g in women and 1.97 g in men) is very similar to the results from a large Swiss study of middle-aged men and women [11]. If applying the regression equations (including sex, BMI and age) from that study on our smaller population, the expected excretion in our study would be 1.4 g in women and 1.9 g in men.

The ICC for 24 h creatinine excretion was 0.64 in both men and women, showing that the variability between individuals was much larger than the intra-individual variability between days. In a study of repeated measurements among 11 non-smoking Chinese men the ICC for 24 h creatinine was 0.54 for samples collected days apart and 0.61 for samples months apart [13]. Variability between individuals is mainly due to muscle mass and age. Variability within individuals could be due to varying consumption of cooked meat, or exercise, between days. In a small experimental study, high intake of cooked meat increased the 24 h excretion of creatinine by 13% [14]. Sometimes, the total variance is expressed as the coefficient of variation (CV), the typical CV has been reported to be about 10% [2], which is similar to our finding of 13%. 

### 4.3. Diurnal Variation of Creatinine in Spot Samples

There was a clear diurnal variation both for creatinine concentrations and creatinine excretion rates. As expected, creatinine concentrations were highest in the more concentrated ON samples and lowest in the samples collected at 12:00 and 14:30 when the urinary flow rate was highest. 

For the creatinine excretion rate the situation was reversed with the lowest excretion rates in ON samples. This is important since the rationale behind adjustment for creatinine concentration assumes the creatinine excretion rate is constant. The lower excretion rate overnight has been reported previously [4]. It is likely that this is because the glomerular filtration rate (GFR) is 20–30% higher during day time than during night, despite the fact that this impact on GFR is partly counteracted by higher tubular secretion of creatinine during the night [15,16]. Additionally, protein and creatine intake from meat contribute to creatinine excretion and occurs during daytime [14]. 

The positive association between urinary flow and creatinine excretion rate has been demonstrated previously [3,17]. Greenberg and Levine examined repeated urine samples over 2–3 24-h periods from 20 individuals and reported that 21% of the variability in creatinine excretion rate was due to urinary flow rate. Araki et al. [17] reported a Spearman correlation coefficient of about 0.4 between creatinine excretion rate and urinary flow rate. This could not be explained only by a lower creatinine excretion rate in ON samples, as the positive correlation between urinary flow and creatinine excretion rate was seen in ON samples as well as in daytime samples [18]. In addition, creatinine excretion rates increased under experimental water loading, which increased urinary flow [19]. This was confirmed in our data, showing an association between urinary flow rate and creatinine excretion rate within individuals also when excluding the ON samples. The impact of urinary flow on creatinine excretion rate in daytime was, however, not strong enough to result in significantly higher creatinine excretion in the middle of the day. The slope of the log-log relationship of 0.24 between these variables in our study was much lower than the slope of 0.67 found by Greenberg and Levine [3]. This shows that further adjustment for urinary flow rate (in addition to creatinine concentration) is complicated. Nevertheless the fact that the creatinine excretion rate is not constant should be acknowledged as a limitation when using creatinine adjustment of a target biomarker as a surrogate for the timed excretion of the biomarker. 

### 4.4. Variability of Creatinine in Spot Samples 

For all spot samples (*n* = 718), taken at any time of the day, within-individual variability was much higher than between-individual variability (ICC about 0.25, Table 3). This is similar to the ICC of 0.25 found by Smolders et al. [12] at repeated sampling in eight individuals but higher than ICC of 0.12 found by Wang et al. [13]. For ON samples, within- and between-individual variabilities were similar, with ICC about 0.5 in our study and comparable to the ICCs found in the Chinese study [13]. Thus, when creatinine adjustment is used for various urine biomarkers, there will be less within-individual variability in the denominator of the ratio (analyte concentration/creatinine concentration) when ON samples are used. For biomarkers with a short half-life and rapid excretion after dietary intake this will be the case also for the numerator. However, in practice, the intra-class correlation of the unadjusted, creatinine-adjusted, and SG-adjusted biomarker concentrations should be compared when they are to be used in epidemiological studies [12,20]. Results comparing unadjusted and creatinine-adjusted samples have been presented for six short-lived substances in 16 individuals [21] and for some metals (arsenic, manganese, nickel and cadmium) among individuals from the general population [12,20]. Adjusting for creatinine provided the highest ICC for all metals and for four out of six of the short-lived substances. The highest ICCs were seen for 24 h sampling time [2,20] and similar finding have been found for urinary polycyclic aromatic hydrocarbon metabolites [22]. In a study where biomarkers were divided into three classes according to residence time the variance ratio (within-individual variance/between-individual variance) decreased with residence time and was lowest for the group of substances with half-time of more than 2 months [23]. Our ICC calculations are based on repeated samples from 60 subjects which is enough to give a valid point estimate of the ICC [24] and a higher number of participants than in some other studies [2,12,22].

### 4.5. Creatinine versus Specific Gravity

About 6% of the samples had creatinine concentrations outside the recommended limits for adjustment of other analytes, while the corresponding figure for specific gravity was 22%. These results are in agreement with the study by Smolders et al. [12]. Similar percentages outside the limits for creatinine concentration were found in studies of workers, including more than 50,000 urine samples [8] and more than 1000 samples [25]. In two very large studies in the general U.S. population (NHANES III and NHANES 2009–2012) about 8% of the samples were very diluted [26,27], slightly higher than the 4% found in our study. The difference between men and women in creatinine concentrations found in our study and others [2,8,26,27,28] is often used as an argument against creatinine adjustment of urinary analytes [29]. As shown in Table 2, there was, however, also a difference in SG, women having about 25% lower SG than men. The high correlation between creatinine concentration and SG (0.87) found in our study has also been seen in other studies, both for 24-h and spot samples [12,25,28]. Spearman correlations between 24 h metal concentrations adjusted for creatinine concentration or SG were high (>0.8) for 18 out of 22 elements [30]. In our opinion both methods of adjustment are acceptable in studies of healthy individuals.

### 4.6. Limitations

First, we did not measure fluid intake, exercise or intake of specific food items on the day of sampling, which is sometimes performed in experimental studies. This is necessary if the impact of these factors on creatinine excretion is examined. However, the present study aimed at assessing normal variability in creatinine excretion, rather than quantifying the relative importance of factors affecting the variability. Second, although the sample size (*n* = 60) was larger than previous studies of creatinine variability in 24 h urine, the study group is still small and homogeneous (only healthy non-smoking individuals) and there is still some uncertainty about variance components. Further studies are warranted.

## 5. Conclusions

For 24 h urine samples, between-individual variability of creatinine excretion is larger than within-individual variability (high ICC). For overnight samples the between- and within-individual variabilities are similar (ICC about 0.5). In daytime spot samples, the within-individual variability dominates (low ICC).

The biobank presented here, which is free to use for other researchers, is especially suitable for analysis of diurnal variation in urine biomarkers (12 samples per subject), but it can also be used for analyses of variability in biomarker concentrations in blood, plasma or red cells, and for associations between concentrations in blood and urine.

## Figures and Tables

**Table 1:** Background factors in 60 subjects participating in the variability biobank.

	All (*n* = 60)		Women (*n* = 31)		Men (*n* = 29)	
	Mean, median (range)	%	Mean, median (range)	%	Mean, median (range)	%
Age	34, 31 (21–64)		35, 31 (21–62)		33, 29 (21–64)	
Body weight (kg)	72, 70 (49–140)		62, 61 (49–80)		83, 78 (65–140)	
BMI (kg/m^2^)	24, 23 (19–44)		23, 22 (19–28)		25, 24 (21–44)	
Rice (meals/week), % ≥ 3	2.3, 2.0 (0–10)	30	1.8, 1.0 (0–6)	19	2.8, 2.0 (0–10)	41
Fish (meals/week), % ≥ 2	1.8, 2.0 (0–7)	52	1.8, 1.5 (0–7)	48	1.9, 2.0 (0–5)	51
Meat (meals/week), % ≥ 6	4.7, 4.5 (0–14)	32	3.8, 4.0 (0–10)	23	5.6, 5.0 (1–14)	41
Amalgam fillings		20 ^a^		9 ^a^		30 ^a^
Exercise (≥2 × 30 min/week)		78		77		79

^a^ Information missing for 10 subjects, 8 women and 2 men.

**Table 2:** Urinary excretion in 24 h for 60 subjects participating in the variability biobank. Mean, median and (range) of individual two-day means (one subject has missing data for one day) are given.

	All (*n* = 60)	Women (*n* = 31)	Men (*n* = 29)
Total urinary volume (L)	1.68, 1.58 (0.84–3.66)	1.69, 1.61 (0.84–2.70)	1.67, 1.53 (0.99–3.66)
Collection time (h)	24, 24 (22–28)	24, 24 (23–28)	24, 24 (22–25)
Urinary flow rate in 24 h (mL/h)	70, 66 (35–152)	70, 68 (35–111)	70, 65 (41–152)
Urine flow rate per body weight in 24 h (ml/kg, h)	1.02, 0.96 (0.38–2.17)	1.15, 1.13 (0.57–2.13)	0.88, 0.79 (0.38–2.17
Creatinine excretion rate (g/24 h)	1.60, 1.51 (0.77–3.99)	1.27, 1.26 (0.77–1.82)	1.96, 1.94 (1.30–3.99)
Creatinine excretion rate (g/24 h/kg body weight)	0.022, 0.023 (0.011–0.036)	0.021, 0.022 (0.012–0.031)	0.024, 0.024 (0.011–0.036)
Creatinine excretion rate (g/h)	0.066, 0.062 (0.032–0.165)	0.052, 0.053 (0.033–0.073)	0.082, 0.080 (0.056–0.165)
Creatinine concentration (g/L)	1.06, 0.94 (0.40–2.26)	0.82, 0.76 (0.40–1.74)	1.31, 1.26 (0.56–2.26)
Specific gravity	1.017, 1.016 (1.008–1.031)	1.015, 1.014 (1.009–1.026)	1.020, 1.019 (1.008–1.031)

**Table 3:** Within- and between-individual variance (with 95% confidence interval (CI)) and intra-class correlation (ICC) for urinary flow rate and creatinine for different sampling times.

		Within-Individual	Between-Individual		
Samples	*n*	Variance (95% CI)	Variance (95% CI)	ICC	Sex ^1^
**24-h**	119				
Flow rate (mL/h)		0.05 (0.03, 0.07)	0.08 (0.05, 0.13)	0.62	ns
Flow rate/kg (mL/kg, h)		0.05 (0.03, 0.07)	0.11 (0.07, 0.13)	0.70	*p* = 0.003
Creatinine conc. (g/L)		0.04 (0.03, 0.06)	0.10 (0.07, 0.16)	0.72	*p* < 0.001
Creatinine excretion rate (g/h)		0.02 (0.01, 0.03)	0.03 (0.02, 0.06)	0.64	*p* < 0.001
**All spot samples**	718				
Flow rate (mL/h) female		0.41 (0.25, 0.47)	0.07 (0.02, 0.12)	0.14	ns
Flow rate (mL/h) male		0.30 (0.26, 0.35)	0.10 (0.03, 0.16)	0.24	
Creatinine conc. (g/L), female		0.33 (0.29, 0.39)	0.09 (0.03, 0.15)	0.22	*p* < 0.0001
Creatinine conc. (g/L), male		0.25 (0.22, 0.29)	0.12 (0.04, 0.19)	0.32	
Creatinine excretion rate (g/h)		0.12 (0.10, 0.13)	0.03 (0.02, 0.06)	0.23	*p* < 0.0001
**Overnight (ON) samples**	119				
Flow rate (mL/h) female		0.22 (0.14, 0.40)	0.18 (0.02, 0.33)	0.44	ns
Flow rate (mL/h) male		0.13 (0.08, 0.24)	0.03 (−0.04, 0.09)	0.16	
Creatinine conc. (g/L), female		0.11 (0.07, 0.19)	0.21 (0.08, 0.35)	0.71	*p* < 0.001
Creatinine conc. (g/L), male		0.09 (0.05, 0.16)	0.05 (−0.01, 0.10)	0.51	
Creatinine excretion rate (g/h)		0.06 (0.04, 0.09)	0.06 (0.03, 0.12)	0.48	*p* < 0.0001

^1^*p*-values for sex as fixed effect in mixed-effect models, ns = not significant.

## Data Availability

Data are available upon request.

## Supplementary Materials

The following are available online at https://www.mdpi.com/1660-4601/18/6/3166/s1. Figure S1: Association between specific gravity and creatinine concentration in 24-h urine (*n* = 60, first sampling day); Figure S2: Median values of volume and flow rate (left axis) and specific gravity (right axis) for 31 women and 29 men in each of two 24-h periods; Figure S3: Median values of creatinine concentration (mg/dL), creatinine excretion rate (mg/h) and urine flow rate (ml/h) in 31 women and 29 men in each of two 24-h periods; Table S1: Blood samples: tubes, volume and numbers stored in the biobank; Table S2: Number of diluted or concentrated urine samples /total numbers; Table S3: Urine data (volume, collection time, flow rate, creatinine concentration, creatinine excretion rate and specific gravity) in 60 subjects on two different days with six collection times each day; Table S4: Urine data (volume, collection time, flow rate, creatinine concentration, creatinine excretion rate and specific gravity) in 31 women on two different days with six collection times each day; Table S5: Urine data (volume, collection time, flow rate, creatinine concentration, creatinine excretion rate and specific gravity) in 29 men on two different days with six collection times each day.
