# Supplementary material for: Variability of Urinary Creatinine in Healthy Individuals
Source: Int J Environ Res Public Health. 2021 Mar 19;18(6):3166. doi: 10.3390/ijerph18063166 (PMC8003281; doi:10.3390/ijerph18063166)
Supplement: Supplementary file 1 [file ijerph-18-03166-s001.pdf]

Supplementary Information: Figures S1, S2 and S3. Tables S1, S2, S3, S4 and S5.

Figure S1. Association between specific gravity and creatinine concentration in 24-hour urine (N=60, first sampling day).

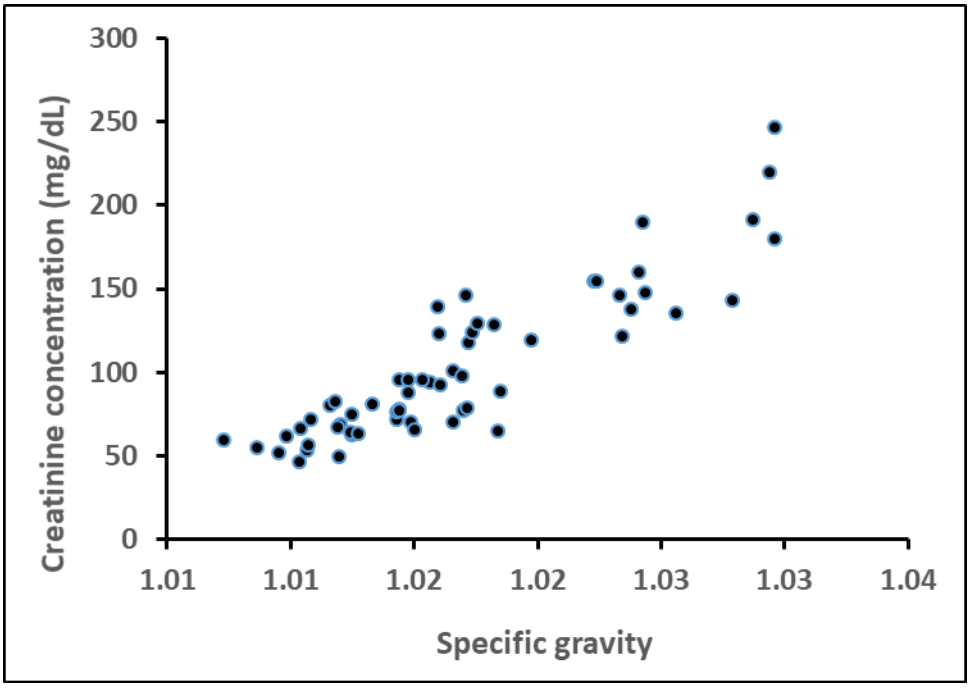

Figure S2. Median values of volume and flow rate (left axis) and specific gravity (right axis) for 31 women and 29 men in each of two 24-h periods.

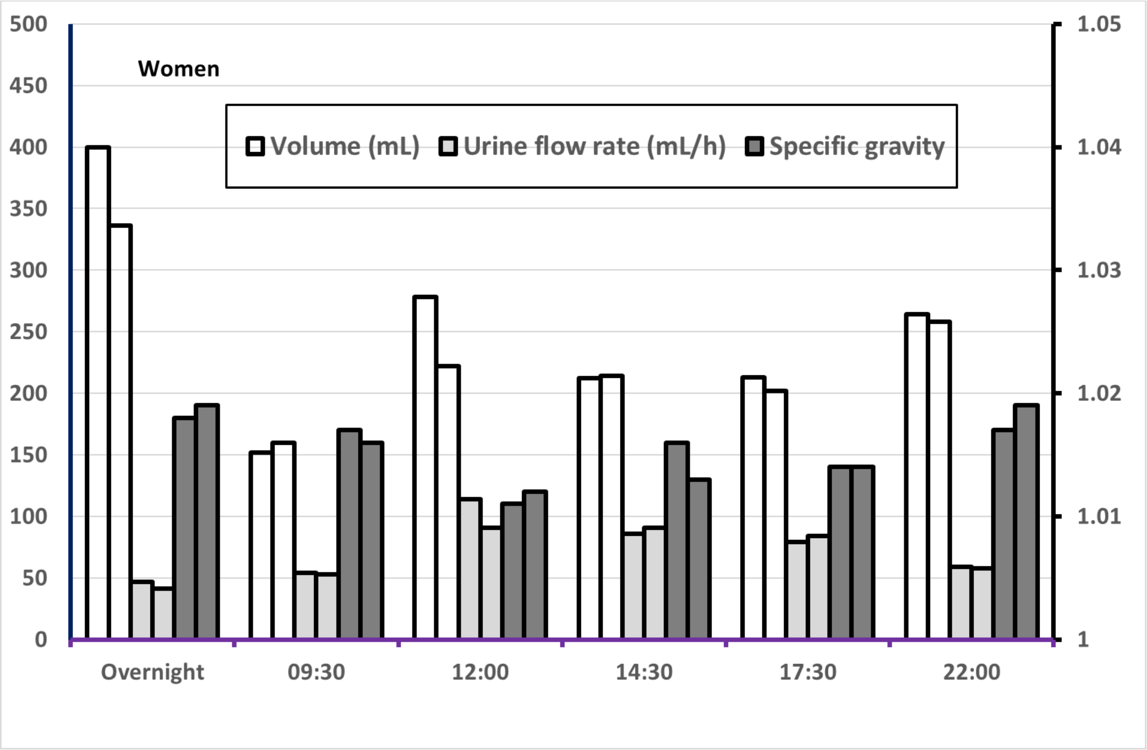

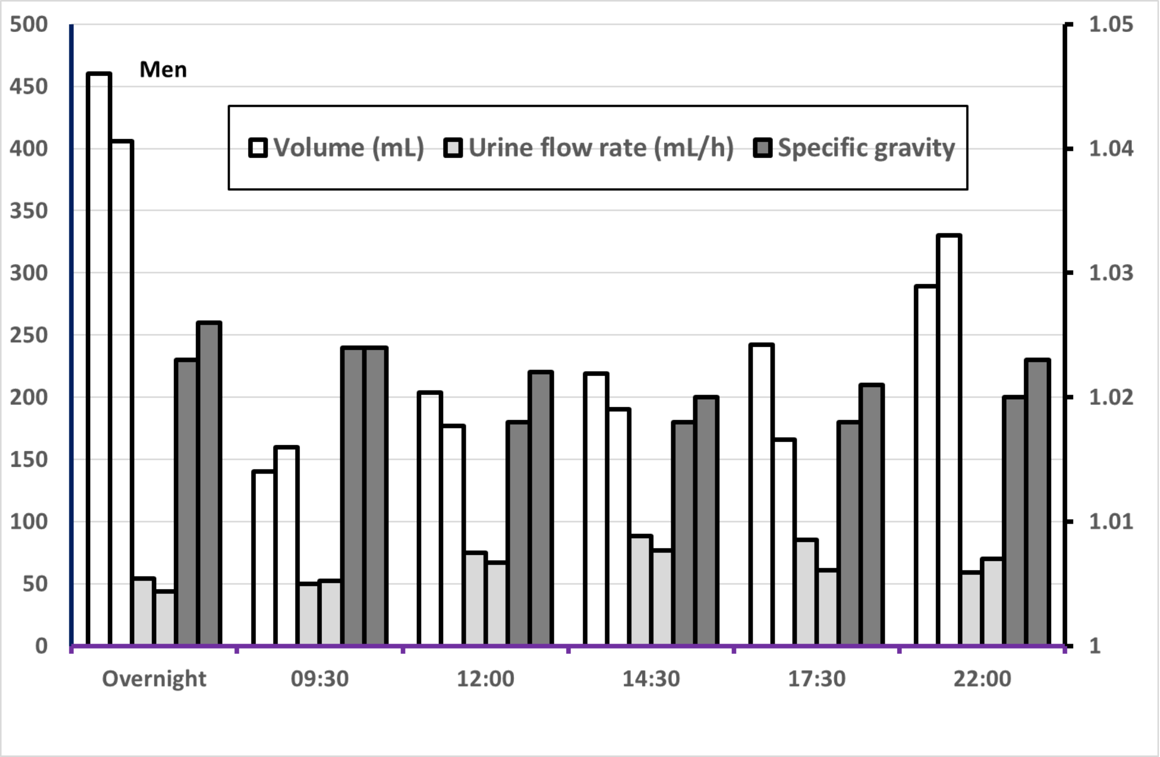

Figure S3. Median values of creatinine concentration (mg/dL), creatinine excretion rate (mg/h) and urine flow rate (ml/h) in 31 women and 29 men in each of two 24-h periods.

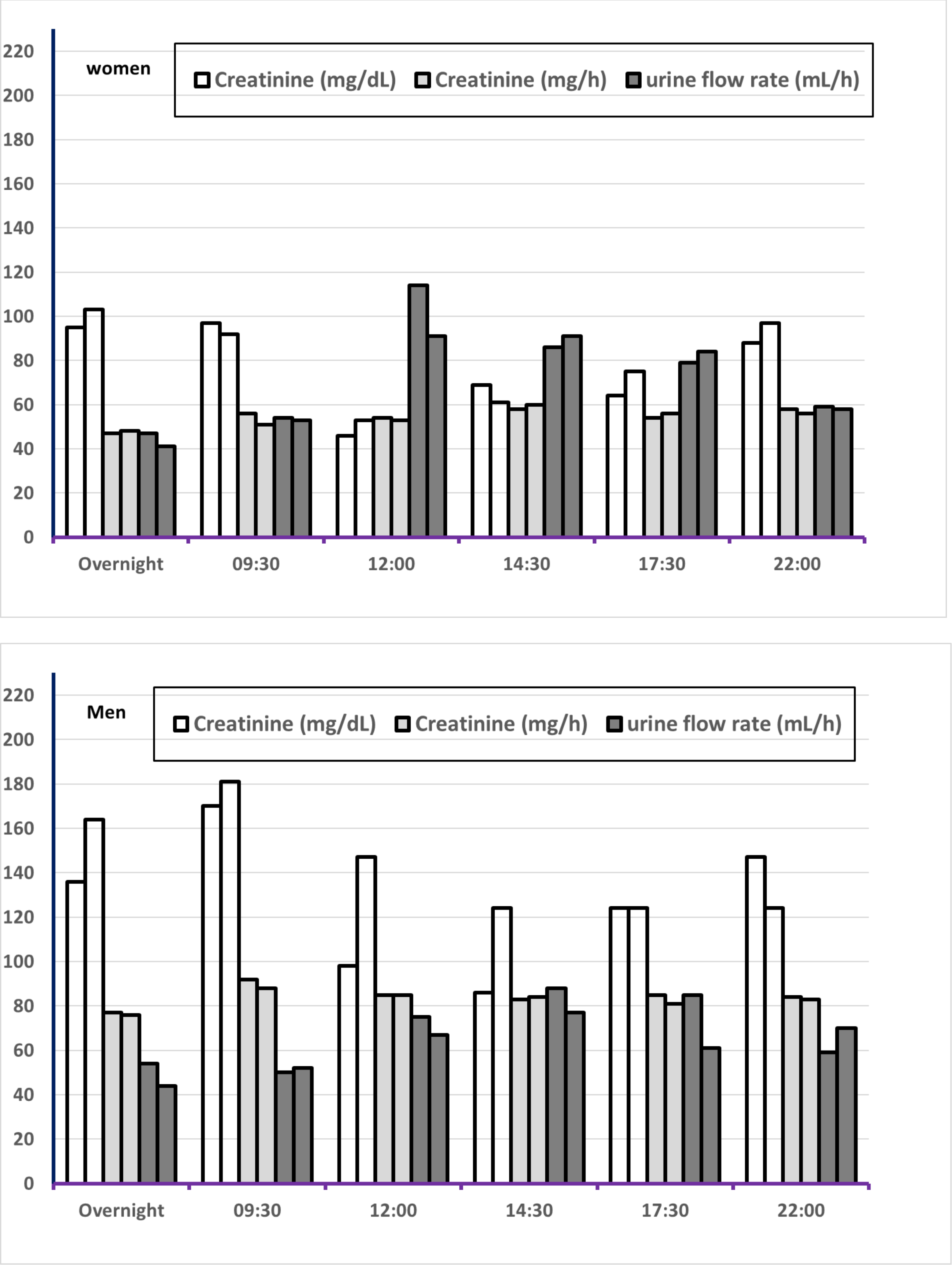

Table S1. Blood samples: tubes, volume and numbers stored in the biobank.

Type of sampling tubes [1]	Volume	Product	Volume received/day, id	Storage tubes[2]	Type of pipettes used	Volume/PP tube	N. Numbers stored in the biobank/day, id
BD 368430<br>Serum tubes without additives	10mL	Serum	~ 4mL	0.5mL PP	Acetone-washed Pasteur Pipettes (Glas)	0.5mL serum	8
VACUETTE 454056<br>Lithium Heparin	4mL	Whole blood	~ 4mL	2mL PP	Acid-washed 0.1-1mL Plastic pipettes	1mL whole blood	1
				0.5mL PP		0.5mL whole blood	6
BD 368886<br>Lithium Heparin	6mL	Heparine plasma	~ 3mL	0.5mL PP	Acid-washed 0.1-1mL Plastic pipettes	0.5mL plasma	5
		Erythrocytes	~ 3mL	0.5mL PP	Acid-washed 0.1-1mL Plastc pipettes	0.5mL erythrocytes	5
BD 367704<br>Coagulation Tube, 3,8%	4.5mL	Citrate plasma	~ 2mL	0.5mL PP	Untreated	0.5mL plasma	4

1) BD (Becton Dickinson, US), Vacuette (Greiner Bio-One North America, Inc). 2) PP= Polypropene tubes (Sarstedt, Nürmbrecht, Germany)

Table S2. Number of diluted or concentrated urine samples /total numbers

times	creatine <0.3 g/L	creatinine > 3 g/L	specific gravity < 1.01	specific gravity >1.03
all	29/718	16/718	106/718	54/718
09:30	5/120	7/120	16/120	17/120
12:00	15/120	2/120	30/120	5/120
14:30	1/120	1/120	21/120	4/120
17:30	7/119	3/119	23/119	7/119
22:00	1/120	1/120	11/120	10/120
overnight	0/119	2/119	5/119	11/119

Table S3. Urine data (volume, collection time, flow rate, creatinine concentration, creatinine excretion rate and specific gravity) in 60 subjects on two different days with six collection times each day.

Variable	Time	Day	N	Mean (STD)	Median	Range (min – max)
Volume mL	9:30	1	60	171 (115)	144	21 – 505
		2	60	183 (127)	160	8 – 618
	12:00	1	60	277 (155)	254	53 – 888
		2	60	250 (162)	201	53 – 717
	14:30	1	60	236 (133)	213	52 – 685
		2	60	252 8167)	200	28 – 872
	17:30	1	59	277 (181)	238	50 – 958
		2	60	226 (139)	185	52 – 728
	22:00	1	60	305 (147)	271	103 – 770
		2	60	332 (217)	294	43 –1027
	Overnight	1	60	445 (194)	429	85 – 1029
		2	59	408 (198)	352	70 – 1020
Collection hours	9:30	1	60	2.65 (0.76)	2.63	0.75 -– 5.50
		2	60	2.72 (0.90)	2.63	1.42 -– 7.42
	12:00	1	60	2.47 (0.36)	2.50	1.25 -– 3.50
		2	60	2.52 (0.35)	2.50	1.63 – 3.50
	14:30	1	60	2.47(0.28)	2.50	1.67 – 3.17
		2	60	2.50 (0.29)	2.50	2.00 – 3.25
	17:30	1	59	2.91 (0.48)	2.98	0.83 – 4.08
		2	60	2.89 (0.53)	3.00	0.67 – 4.00
	22:00	1	60	4.57 (0.58)	4.50	3.50 – 7.00
		2	60	4.46 (0.61)	4.50	2.42 – 6.92

	Overnight	1	60	8.83 (1.00)	8.88	6.17 – 11.0
		2	59	8.85 (0.91)	8.92	6.92 – 11.0
Urinary flow rate ml/h	9.30	1	60	63.8 (40.8)	52.1	16.8 – 178
		2	60	69.4 (50.0)	52.6	5.1 – 242
	12:00	1	60	116.2 (69.1)	101.8	20.5 – 300
		2	60	102.8 (70.6)	74.6	18.3 – 308
	14:30	1	60	96.8 (59.4)	86.8	24.7 – 357
		2	60	101.3 (67.2)	80.4	11.6 – 374
	17:30	1	59	199.0 (72.9)	79.9	14.3 – 408
		2	60	81.7 (51.3)	61.8	15.2 – 224
	22:00	1	60	67.4 (34.2)	58.7	24.7 – 192
		2	60	75.1 (47.9)	64.9	9.6 – 228
	Overnight	1	60	51.1 (23.1)	48.7	9.3 – 120
		2	59	46.6 (22.6)	41.8	6.95 – 116
Creatinine g/L	9:30	1	60	1.51 (1.02)	1.30	0.23 – 5.88
		2	60	1.46 (1.00)	1.36	0.10 – 5.88
	12:00	1	60	0.88 (0.73)	0.66	0.16 – 4.07
		2	60	1.03 (0.71)	0.87	0.18 – 3.28
	14:30	1	60	1.02 (0.68)	0.83	0.31 – 3.62
		2	60	0.98 (0.52)	0.84	0.26 – 2.38
	17:30	1	59	1.04 (0.65)	0.90	0.24 – 3.05
		2	60	1.18 (0.70)	1.13	0.23 – 3.51
	22:00	1	60	1.21 (0.57)	1.06	0.33 – 2.38
		2	60	1.20 (0.71)	1.02	0.29 – 3.28
	Overnight	1	60	1.37 (0.67)	1.24	0.38 – 3.05
		2	59	1.48 (0.73)	1.47	0.37 – 4.41

Creatinine g/h	9:30	1	60	0.071 (0.030)	0.066	0.014 – 0.190
		2	60	0.071 (0.038)	0.066	0.013 – 0.224
	12:00	1	60	0.070 (0.026)	0.063	0.022 – 0.171
		2	60	0.073 (0.040)	0.064	0.017 – 0.258
	14:30	1	60	0.074 (0.028)	0.069	0.023 – 0.169
		2	60	0.082 (0.059)	0.067	0.012 – 0.465
	17:30	1	59	0.074 (0.035)	0.067	0.021 – 0.221
		2	60	0.071 (0.033)	0.060	0.026 – 0.246
	22:00	1	60	0.071 (0.027)	0.068	0.011 – 0.175
		2	60	0.069 (0.033)	0.064	0.022 – 0.224
	Overnight	1	60	0.060 (0.024)	0.058	0.021 – 0.144
		2	59	0.058 (0.022)	0.054	0.013 – 0.112
SG	9:30	1	60	1.021 (0.008)	1.022	1.006 – 1.040
		2	60	1.020 (0.009)	1.021	1.004 – 1.042
	12:00	1	60	1.015 (0.008)	1.013	1.004 – 1.040
		2	60	1.017 (0.008)	1.018	1.004 – 1.031
	14:30	1	60	1.017 (0.008)	1.017	1.005 – 1.040
		2	60	1.017 (0.007)	1.018	1.006 – 1.034
	17:30	1	59	1.017 (0.008)	1.016	1.006 – 1.033
		2	60	1.018 (0.008)	1.018	1.006 – 1.035
	22:00	1	60	1.020 (0.007)	1.019	1.008 – 1.035
		2	60	1.019 (0.008)	1.020	1.006 – 1.034
	Overnight	1	60	1.020 (0.007)	1.020	1.006 – 1.034
		2	59	1.021 (0.008)	1.022	1.008 – 1.039

Table S4. Urine data (volume, collection time, flow rate, creatinine concentration, creatinine excretion rate and specific gravity) in 31 women on two different days with six collection times each day.

Variable	Time	Day	N	Mean (STD)	Median	Range (min – max)
Volume mL	9:30	1	31	191 (127)	152	43 – 505
		2	31	194 (232)	160	8 – 618
	12:00	1	31	292 (123)	278	92 – 612
		2	31	260 (152)	222	53 – 572
	14:30	1	31	229 (115)	212	52 – 527
		2	31	249 (130)	214	28 – 535
	17:30	1	30	277 (181)	213	50 – 768
		2	31	243 (153)	202	52 – 728
	22:00	1	31	308 (136)	264	103 – 623
		2	31	282 (147)	258	80 – 597
	Overnight	1	31	452 (232)	400	85 – 1029
		2	31	391 (213)	336	70 – 1020
Collection hours	9:30	1	31	2.86 (0.70)	2.83	0.75 – 5.50
		2	31	2.77 (0.65)	2.83	1.58 – 4.00
	12:00	1	31	2.43 (0.46)	2.48	1.25 – 3.50
		2	31	2.54 (0.36)	2.50	1.63 – 3.50
	14:30	1	31	2.55 (0.29)	2.50	1.92 – 3.17
		2	31	2.51 (0.26)	2.50	2.00 – 3.08
	17:30	1	30	2.85 (0.44)	2.83	2.00 – 4.08
		2	31	2.88 (0.50)	3.00	1.33 – 3.58
	22:00	1	31	4.62 (0.58)	4.53	3.50 – 6.75
		2	31	4.37 (0.60)	4.50	2.42 – 6.07

	Overnight	1	31	8.90 (0.95)	9.00	7.00 – 11.0
		2	31	8.92 (0.84)	8.92	7.00 – 11.0
Urinary flow rate ml/h	9.30	1	31	68.3 (46.7)	53.8	17.2 – 178
		2	31	69.7 (51.2)	53.0	5.05 – 204
	12:00	1	31	128.4 (70.2)	113.6	36.8 – 300
		2	31	107.3 (69.7)	91.0	18.3 – 308
	14:30	1	31	89.5 (43.5)	85.6	26.0 – 192
		2	31	99.8 (52.8)	91.4	11.6 – 238
	17:30	1	30	97.5 (64.0)	79.1	22.1 – 271
		2	31	87.9 (55.2)	83.9	15.2 – 224
	22:00	1	31	67.3 (30.6)	58.7	24.7 – 159
		2	31	64.6 (30.9)	58.1	17.8 – 133
	Overnight	1	31	51.8 (27.9)	46.8	9.3 – 120
		2	31	44.5 (23.9)	41.4	6.95 – 104
Creatinine g/L	9:30	1	31	1.10 (0.66)	0.97	0.23 – 2.38
		2	31	1.21 (1.16)	0.92	0.10 – 5.88
	12:00	1	31	0.58 (0.36)	0.46	0.20 – 1.47
		2	31	0.82 (0.67)	0.53	0.18 – 3.28
	14:30	1	31	0.86 (0.49)	0.69	0.31 – 2.49
		2	31	0.74 (0.36)	0.61	0.26 – 1.81
	17:30	1	30	0.84 (0.58)	0.64	0.24 – 2.26
		2	31	0.91 (0.53)	0.75	0.23 – 2.15
	22:00	1	31	0.97 (0.49)	0.88	0.33 – 2.15
		2	31	1.07 (0.61)	0.97	0.36 – 2.60
	Overnight	1	31	1.12 (0.59)	0.95	0.38 – 2.71
		2	31	1.34 (0.88)	1.03	0.37 – 4.41

Creatinine g/h	9:30	1	31	0.053 (0.020)	0.056	0.014 – 0.112
		2	31	0.049 (0.018)	0.051	0.013 – 0.078
	12:00	1	31	0.056 (0.013)	0.054	0.022 – 0.087
		2	31	0.060 (0.032)	0.053	0.017 – 0.196
	14:30	1	31	0.062 (0.016)	0.058	0.031 – 0.114
		2	31	0.060 (0.019)	0.060	0.012 – 0.128
	17:30	1	30	0.057 (0.018)	0.054	0.021 – 0.109
		2	31	0.057 (0.015)	0.056	0.026 – 0.114
	22:00	1	31	0.056 (0.017)	0.058	0.011 – 0.100
		2	31	0.055 (0.016)	0.056	0.022 – 0.095
	Overnight	1	31	0.047 (0.014)	0.047	0.021 – 0.079
		2	31	0.044 (0.014)	0.048	0.013 – 0.067
SG	9:30	1	31	1.018 (0.008)	1.017	1.006 – 1.031
		2	31	1.018 (0.010)	1.016	1.004 – 1.042
	12:00	1	31	1.012 (0.005)	1.011	1.004 – 1.024
		2	31	1.015 (0.008)	1.012	1.005 – 1.030
	14:30	1	31	1.016 (0.006)	1.016	1.005 – 1.028
		2	31	1.015 (0.006)	1.013	1.007 – 1.028
	17:30	1	30	1.015 (0.007)	1.014	1.006 – 1.032
		2	31	1.015 (0.007)	1.014	1.006 – 1.031
	22:00	1	31	1.019 (0.007)	1.017	1.008 – 1.035
		2	31	1.018(0.007)	1.019	1.007 – 1.030
	Overnight	1	31	1.018 (0.007)	1.018	1.006 – 1.032
		2	31	1.019 (0.008)	1.016	1.008 – 1.034

Table S5. Urine data (volume, collection time, flow rate, creatinine concentration, creatinine excretion rate and specific gravity) in 29 men on two different days with six collection times each day.

Variable	Time	Day	N	Mean (STD)	Median	Range (min – max)
Volume mL	9:30	1	29	150 (99.7)	140	21 – 400
		2	29	170 (109)	160	64 – 565
	12:00	1	29	261 (184)	204	53 – 888
		2	29	239 (173)	177	68 – 717
	14:30	1	29	243 (151)	219	60 – 685
		2	29	256 (202)	190	70 – 872
	17:30	1	29	277 (185)	242	50 – 958
		2	29	208 (123)	166	53 – 483
	22:00	1	29	302 (159)	289	113 – 770
		2	29	384 (265)	330	43 – 1027
	Overnight	1	29	437 (147)	460	167 – 692
		2	28	428 (183)	406	157 – 913
Collection hours	9:30	1	29	2.44 (0.77)	2.50	0.75 – 4.08
		2	29	2.67 (1.11)	2.33	1.42 – 7.42
	12:00	1	29	2.51 (0.22)	2.50	2.08 – 3.17
		2	29	2.50 (0.34)	2.47	1.97 – 3.42
	14:30	1	29	2.39 (0.24)	2.50	1.67 – 2.67
		2	29	2.48 (0.33)	2.50	2.00 – 3.25
	17:30	1	29	2.98 (0.51)	3.08	0.83 – 3.50
		2	29	2.90 (0.58)	3.00	0.66 – 4.00
	22:00	1	29	4.54 (0.59)	4.50	3.92 – 7.00
		2	29	4.54 (0.62)	4.50	2.67 – 6.92

	Overnight	1	29	8.77 (1.07)	8.83	6.17 – 10.75
		2	28	8.77 (0.99)	8.80	6.92 – 11.0
Urinary flow rate ml/h	9.30	1	29	59.1 (33.5)	50.3	16.8 – 155
		2	29	69.1 (49.5)	52.2	23.6 – 242
	12:00	1	29	103.3 (66.9)	75.2	20.5 – 290
		2	29	98.1 (72.5)	67.3	27.4 – 297
	14:30	1	29	104.5 (72.7)	88.0	24.7 – 357
		2	29	102.9 (80.7)	76.8	29.0 – 373
	17:30	1	29	100.5 (82.2)	85.4	14.2 – 408
		2	29	75,1 (47.0)	61.3	24.3 – 198
	22:00	1	29	67.6 (38.3)	58.8	28.9 – 192
		2	29	86.3 (59.6)	69.7	9.56 – 228
	Overnight	1	29	50.3 (16.9)	53.8	18.9 – 81.4
		2	28	49.0 (21.1)	44.0	17.1 – 116
Creatinine g/L	9:30	1	29	1.94 (1.16)	1.70	0.54 – 5.88
		2	29	1.71 (0.74)	1.81	0.55 – 3.73
	12:00	1	29	1.20 (0.89)	0.98	0.16 – 4.07
		2	29	1.26 (0.69)	1.47	0.28 – 2.49
	14:30	1	29	1.19 (0.82)	0.86	0.31 – 3.62
		2	29	1.23 (0.54)	1.24	0.32 – 2.38
	17:30	1	29	1.25 (0.66)	1.24	0.34 – 3.05
		2	29	1.46 (0.77)	1.24	0.29 – 3.51
	22:00	1	29	1.46 (0.54)	1.47	0.46 – 2.38
		2	29	1.34 (0.78)	1.24	0.29 – 3.28
	Overnight	1	29	1.64 (0.67)	1.36	0.55 – 3.05
		2	29	1.63 (0.51)	1.64	0.86 – 2.49

Creatinine g/h	9:30	1	29	0.090 (0.030)	0.092	0.036 – 0.190
		2	29	0.096 (0.039)	0.088	0.054 – 0.224
	12:00	1	29	0.085 (0.029)	0.085	0.027 – 0.171
		2	29	0.088 (0.042)	0.085	0.035 – 0.258
	14:30	1	29	0.087 (0.031)	0.083	0.023 – 0.169
		2	29	0.106 (0.077)	0.084	0.030 – 0.465
	17:30	1	29	0.091 (0.039)	0.085	0.027 – 0.222
		2	29	0.087 (0.041)	0.081	0.036 – 0.246
	22:00	1	29	0.086 (0.028)	0.084	0.032 – 0.175
		2	29	0.084 (0.040)	0.083	0.027 – 0.224
	Overnight	1	29	0.075 (0.023)	0.077	0.034 – 0.144
		2	28	0.073 (0.019)	0.076	0.033 – 0.112
SG	9:30	1	29	1.024 (0.007)	1.024	1.009 – 1.400
		2	29	1.023 (0.008)	1.024	1.008 – 1.038
	12:00	1	29	1.018 (0.010)	1.018	1.004 – 1.040
		2	29	1.019 (0.009)	1.022	1.004 – 1.031
	14:30	1	29	1.019 (0.009)	1.018	1.005 – 1.040
		2	29	1.020 (0.007)	1.020	1.006 – 1.034
	17:30	1	29	1.019 (0.008)	1.018	1.006 – 1.033
		2	29	1.021 (0.008)	1.021	1.006 – 1.035
	22:00	1	29	1.021 (0.007)	1.020	1.008 – 1.033
		2	29	1.020 (0.009)	1.023	1.006 – 1.034
	Overnight	1	29	1.022 (0.007)	1.023	1.007 – 1.034
		2	28	1.024 (0.007)	1.026	1.012 – 1.039
